# Availability, Affordability, Awareness, Preferences and Nutritional Impact of Biofortified Crops in Nigeria

**DOI:** 10.3390/nu17061036

**Published:** 2025-03-15

**Authors:** Petya Atanasova, Samrat Singh, Adedotun Adebayo, Folashade Adekunle, Abimbola Adesanmi

**Affiliations:** 1 Department of Metabolism, Digestion and Reproduction, Faculty of Medicine, Imperial College London, London SW7 2AZ, UK; 2 School of Public Health, Faculty of Medicine, Imperial College London, London W12 0BZ, UK; 3 Buddymi Tech Nigeria Ltd., Abuja 900211, Nigeria; 4 Partnership for Child Development, Imperial College London—Nigeria Country Office, Abuja 900211, Nigeria

**Keywords:** micronutrient deficiencies, Nigeria, biofortification, availability, affordability, preferences, school meals

## Abstract

**Background:** Nigeria has one of the highest prevalences of micronutrient deficiencies (MNDs) globally. Biofortification is a sustainable and cost-effective intervention to reduce MNDs. We investigated the current availability, affordability, individual perceptions and preferences regarding biofortified crops in three states in Nigeria (Enugu, Ogun and Kaduna). We investigated potential dietary quality improvements by modifying standardized school meals with biofortified crops. **Methods:** We conducted a field market survey, key informant interviews and a simulation study. The availability and prices of biofortified crops from 36 preselected markets were systematically recorded. Consumers and sellers were interviewed regarding their awareness of and preferences for biofortified crops. The inclusion of biofortified foods in weekly school meals was simulated to evaluate dietary quality improvements and costs. Three key informant interviews were conducted to understand the scalability of biofortified crops. Qualitative and quantitative techniques were employed in the data analysis. **Results:** Overall, 39% (total of n = 730) of the recorded crops were identified as biofortified. Biofortified cassava, sweet potatoes and millet were more expensive compared to non-biofortified equivalents. Moreover, 30% (total of n = 730) of the consumers could visually differentiate between the crops, 14% were aware that they were biofortified and 10% preferred biofortified options. The inclusion of biofortified foods in traditional school meals led to higher levels of vitamin A, zinc and iron. The key informant interviews highlighted that scaling biofortification is challenging, requiring individual behavioral change, significant investments in educational campaigns and improvements in supply and demand. **Conclusions:** The current state of biofortification has limited reach due to limited availability, affordability and consumer preferences.

## 1. Introduction

Micronutrient deficiencies (MNDs), also known as “hidden hunger”, are a critical public health challenge with lifelong and intergenerational consequences [1]. The impact of MNDs on children is associated with cognitive impairment, birth defects, blindness, increased susceptibility to infections, decreased school performance and an increased likelihood of non-communicable diseases such as diabetes and cardiovascular disease [1,2,3,4]. Children are more vulnerable to MNDs due to their higher micronutrient requirements [1].

Globally, a pooled analysis of individual-level biomarker data on the micronutrient status from nationally representative, population-based surveys estimates that over half of preschool-aged children have at least one micronutrient deficiency [5]. The prevalence of MNDs is particularly high in Sub-Saharan Africa, with Nigeria having the highest burden, with an estimated 17 million undernourished children [6]. Estimates from Nigeria’s National Micronutrient Survey in 2021 indicate that 54.1% of children under five are deficient in vitamin A, which is associated with impaired growth, eye damage and an increased risk of infections; 35.2% are deficient in zinc, which is associated with impaired health and cognitive development; and 9.6% are deficient in iron, which is associated with anemia, weakness and impaired psychomotor and mental development [6]. Among adolescent girls (aged 10–14), it was estimated that 31.7% are deficient in vitamin A, 33.5% are deficient in zinc and 3.1% are deficient in iron [6]. Adequate nutrition, especially during early childhood and adolescence, is paramount for optimal growth, health and development.

Healthy diets in Nigeria were estimated to be unaffordable for three in 10 people in 2019 [7]. Disruptions to safety net programs and rapid food price inflation have severe and lifelong consequences for millions of people across the world, exacerbating already high levels of MNDs [8]. Typically, as a response to the unaffordability of healthy diets, households reduce the consumption of nutrient-dense foods and increase the consumption of cheaper foods, which are perceived as more filling but are low in essential nutrients [9,10]. The COVID-19 pandemic and the ongoing Ukraine crisis are emphatic reminders of the urgent need to develop sustainable and resilient food systems.

In a declaration in 2022, the African Union underpinned the importance of agricultural food-based approaches such as biofortification to improve the nutritional status in the population [11]. Biofortification is the process of increasing the nutrient density of food crops through conventional plant breeding, modern biotechnology and agronomic practices, while maintaining crop properties and productivity [12]. Given that biofortification enhances the inherent nutritional properties of food, it can offer a sustainable solution that is integrated with local food systems [13]. This approach can be particularly advantageous in reaching remote rural populations that primarily rely on farming and might not benefit from industrial large-scale fortification efforts [13].

Evidence from both efficacy studies and effectiveness trials demonstrates the positive results of biofortification. For example, a review of the evidence from efficacy studies indicates that iron crops (namely iron bean and iron pearl millet) and vitamin A crops (namely orange sweet potato, vitamin A maize and vitamin A cassava) are effective in reducing iron and vitamin A deficiencies in target populations [9]. Zinc-biofortified wheat starch has also been found to increase total zinc absorption by 30–70% [14,15]. Specific to Nigeria, a randomized controlled trial found that preschool children (3–5 years old) who consumed vitamin A-biofortified yellow cassava twice daily over six months had significantly higher serum retinol concentrations compared to the control group, who consumed white non-biofortified cassava [16]. Similar findings were observed in an effectiveness study in rural Uganda, where biofortified β-carotene-rich orange sweet potato was implemented through a community-based approach over two years, and the prevalence of inadequate vitamin A intake was reduced by 30% among children [17].

The government of Nigeria has achieved initial progress by explicitly defining biofortification as a key strategy to address MNDs [18]. Thus, in 2010, biofortification was launched in Nigeria to tackle vitamin A deficiencies, initially through vitamin A-biofortified cassava; a few years later, vitamin A orange sweet potatoes and vitamin A maize were released [19]. By 2020, an estimated 1.76 million smallholder farming families were growing vitamin A cassava and 756,000 were growing vitamin A maize [19].

Although biofortified crops were introduced in Nigeria over a decade ago, studies suggest that their uptake is limited. The National Micronutrient Survey conducted in 2021 found that only 3%, 5% and 14% of the respondents reported consuming biofortified yellow cassava, orange-fleshed sweet potato and orange maize, respectively, in the last 30 days [6]. While there can be multiple reasons for low intake across production and consumption dimensions, it is important to understand the state of biofortified foods in the market and the perceptions of traders and consumers, the impact on local diets and the cost implications, as biofortified crops can be an important pathway to improve diets and, more specifically, address MND deficiencies in Nigeria and beyond.

In this paper, we aim to analyze these issues based on primary data collection and food basket simulations in three states in Nigeria (Enugu, Kaduna and Ogun). Namely, we explore the availability and affordability of biofortified crops in the market compared to non-biofortified equivalent crops. In addition, we investigate whether sellers and customers are aware of biofortified crops, whether they can visually differentiate between biofortified and non-biofortified crops and their preferences. Furthermore, we simulate the changes in the nutritional composition of local meals if biofortified crops are introduced, using standardized school meals with specific nutrient targets as a case study. Lastly, we discuss the cost implications of the inclusion of biofortified crops in standardized school meals.

## 2. Materials and Methods

This study involved three different methodological components: (1) a field market survey, (2) key informant interviews and (3) a food basket simulation study. The field market survey and key informant interviews were conducted to assess the availability and affordability and consumers’ and sellers’ preferences and perceptions of biofortified foods. The simulation study was conducted to assess how the inclusion of biofortified foods in local meals affects the dietary quality of meals, using standardized school meals with specific nutrient targets as a case study.

### 2.1. Field Market Survey Methodology

A field market survey about biofortified crops was conducted in 2023 in three states in Nigeria, namely Kaduna, Ogun and Enugu. Trained data collectors obtained information by observation about the availability and affordability of biofortified crops and their non-biofortified equivalents while systematically walking through pre-selected markets. Availability was recorded in binary form as yes/no, and, if yes, the total amounts of the crops were recorded. Affordability was recorded by checking the price of the biofortified crop per unit. In addition, the trained data collectors gathered responses to predesigned questions from random customers and sellers at the markets. Namely, the sellers and consumers were asked whether they were aware of biofortified crops and if they could visually differentiate biofortified crops from non-biofortified equivalent crops. Moreover, consumers were asked to report on whether they preferred biofortified or non-biofortified products (the full questionnaire can be found in Supplementary Materials Section S1.1). Sellers were also asked about the typical availability of the crops, with the following options to select from: rarely available, always available, available only in season. The trained data collectors filled in the questionnaire using the Epicollect 5 tool. Data were collected regarding the four major biofortified crops available in Nigeria, namely vitamin A cassava, vitamin A maize, iron pearl millet and orange-fleshed sweet potato.

A multistage sampling approach was employed to select study sites and participants. Firstly, we purposefully selected three states (Kaduna, Enugu and Ogun) and two local government areas (LGAs) per senatorial zone for each state to ensure the geographic and economic representation of the different regions of Nigeria. In Kaduna State, the following LGAs were visited: Lere, Chukun, Zango Kataf, Kauru, Markafi and Kaduna North. In Ogun State, the LGAs visited were Abeokuta North, Imeko Afon, Odeda, Ado Odo, Sagamu and Ijebu North. In Enugu State, the LGAs visited were Enugu North, Nkanu East, Nsukka, Udenu, Aniniri and Udi. Secondly, using a random stratified sampling approach, for each LGA, one urban and one rural market were randomly selected, leading to a total of 12 markets visited per state. A minimum of five randomly selected stalls were visited in each of the markets. Lastly, within each selected area and market, sellers and consumers were randomly selected to participate in the survey.

### 2.2. Geographical and Economic Context

The choice of the three states, Kaduna, Ogun and Enugu, was due to their diverse geographical, economic and agricultural landscapes, offering a comprehensive view of the different regions of the country.

Kaduna State is located in the northwestern part of Nigeria and has a competitive advantage in agriculture due to its vast arable and well-watered land. In total, 38.09% of the state’s GDP comes from agriculture, and 42.4% of the population is actively engaged in agriculture-related activities [20]. In addition, the state is a leading producer of grains, such as maize and sorghum, in Nigeria [20]. Other staples and high-value crops grown in the state include sweet potato, millet, cassava and cotton [20]. The MND levels in Kaduna State are some of the highest in the country, with stunting being at 61.3%, while the national stunting prevalence was reported at 36.1% in 2018 [21]. Furthermore, in Kaduna State, the proportion of children who met the minimum dietary diversity requirement in 2019 was 28.0% [22].

Enugu State is situated in the southeastern part of Nigeria, and about 58.45% of the population live in rural areas, where farming is the most economically viable source of income [23]. In Enugu State, the prevalence of stunting and underweight was 25.0% and 39.8%, respectively, in 2018, which was higher than the reported national prevalence [22].

Ogun State is located in the southwestern region, and 74% of its land is arable and 29% of it is cultivated. Its advantage is that it is geographically close to markets in Lagos State and the Economic Community of West African States, making it suitable for food export. MNDs are also prevalent in Ogun State, with stunting and underweight prevalences being 32.5% and 17.7%, respectively, in 2017 [22]. Both Enugu and Ogun State are in the southern part of Nigeria, which experiences some of the highest rainfall, making them suitable for the production of cassava, sweet potato, yam and cocoyam [24].

Therefore, the selection of Kaduna, Enugu and Ogun was strategic, aiming to reflect the geographical and economic diversity of Nigeria.

### 2.3. Key Informant Interviews

A stakeholder analysis was carried out by in-country collaborators to identify the key actors in biofortification processes and activities in Nigeria, based on their active involvement and participation. The identified actors were HarvestPlus, GAIN and TechnoServe, who we subsequently invited for open-ended exploratory interviews. The interviews (n = 3) were conducted online by three PCD interviewers. The stakeholders were generally probed to discuss topics related to biofortification production costs, factors driving the higher prices of biofortified crops compared non-biofortified equivalents, farmer and consumer acceptability and awareness of biofortified crops and challenges in scaling up biofortification in Nigeria. The content of the interview questions was compiled by one researcher and reviewed by two other researchers (full set of questions available in Supplementary Materials Section S1.2). Each interview lasted about 1 h. Content analysis was undertaken on the information collected through the interviews to supplement the findings of the field market survey. Therefore, in Section 3, we narratively report on the findings from the field market survey and supplement this with insights from the key informant interviews.

### 2.4. Simulation Study Methodology

School meals for each state are developed in participatory workshops with local dishes and ingredients and are standardized with specific nutrient targets. They reflect a realistic food basket that is consumed locally and provide a credible basis on which to understand changes in nutrient quality should biofortified foods be introduced. In consultation with state school feeding management, we systematically identified the optimal means of modifying school menus to introduce biofortification in an acceptable and appropriate way for the local cuisine (predesigned weekly school meals per state can be found in Supplementary Materials S2, Tables S1–S3, with details of which meal is suitable for the introduction of biofortification). All biofortified crops readily available in the market in Nigeria were considered for the modification of the school meals: orange-fleshed sweet potato, vitamin A maize, vitamin A cassava and iron pearl millet. For the three states (Ogun, Kaduna and Enugu), we carefully considered the school meal plan for each day and discussed the most optimal means of modifying the meals to introduce biofortification. For each meal, four options were considered: (1) substituting an ingredient with a biofortified equivalent, (2) adding a biofortified product to the existing meal, (3) completely replacing the meal with an alternative option that contained a biofortified product or (4) leaving the meal unchanged. The preferred approach, where feasible, was the first option, i.e., to identify a suitable biofortified substitute. For example, in Enugu State, the original sweet potato and yam ingredients were substituted with biofortified orange-fleshed sweet potato. When substitution was not possible, the addition of a biofortified product was considered. For example, in Ogun State, to the original bean porridge and fish meal, we added orange-fleshed sweet potato. In some cases, neither substitution nor addition was appropriate and therefore the full meal was changed to a similar alternative meal that could include a biofortified product. For example, in Enugu State, the original meal okpa (Bambara nut) with dried fish was fully changed to a local meal called abacha, which included vitamin A cassava. Similarly, in Kaduna State, the original meal of jollof rice and beans was fully exchanged for a vitamin A maize and beans meal, which used a mix of locally produced grains instead of rice.

The macro- and micronutrients estimated for each meal included energy (kcal), protein (g), fat (g), vitamin A (µg), iron (mg) and zinc (mg). Additionally, we calculated the percentage of the recommended nutrient intake (RNI) provided by each meal, based on the following RNI values: energy = 1871 kcal, protein = 58 g, fat = 17 g, vitamin A = 475 µg, iron = 8 mg and zinc = 5 mg. Nigeria’s food composition table was used to calculate the macro- and micronutrients for each ingredient that was not biofortified. For the biofortified ingredients, the macro- and micronutrient estimations were based on the values provided by HarvestPlus (*Iron Pearl Millet*. HarvestPlus. https://www.harvestplus.org/crop/iron-pearl-millet/ [Accessed 5th September 2024]; *Vitamin A Cassava*. HarvestPlus. https://www.harvestplus.org/crop/vitamin-a-cassava/ [Accessed 5th September 2024]; *Vitamin A Orange Sweet Potato*. HarvestPlus. https://www.harvestplus.org/crop/vitamin-a-sweet-potato/ [Accessed 5th September 2024]; *Vitamin A Maize*. HarvestPlus. https://www.harvestplus.org/crop/vitamin-a-maize/ [Accessed 5th September 2024]).

Lastly, we estimated the cost implications of the inclusion of biofortified crops in school meals using information about the prices of biofortified crops and their non-biofortified equivalents from the field market survey.

### 2.5. Data Analysis

Quantitative data were analyzed using Excel and StataMP 18 to obtain frequencies, percentages and means. Qualitative data were analyzed using narrative synthesis [25].

## 3. Results

### 3.1. Availability and Affordability of Biofortified Crops

#### 3.1.1. Availability

A total of N = 730 crops were recorded (N = 249 in Enugu, N = 134 in Kaduna and N = 347 in Ogun) (Table 1). Half of the recorded crops were maize (53,49%), a third were sweet potatoes (36.34%), cassava constituted 10% and millet constituted 1.16%. All four crops (cassava, maize, millet, sweet potato) were observed in Enugu State, three of the crops (cassava, maize, sweet potato) were observed in Ogun State, and, in Kaduna State, only maize was observed.

In total, 39% of the recorded crops were identified as biofortified (17.27% in Enugu, 100% in Kaduna, 0.29% in Ogun State). The most common biofortified crop was maize (86.52% of all biofortified crops), followed by sweet potatoes (10.67%), millet(1.69%) and cassava (1.12%). The most common nutrient in the biofortified crops was vitamin A, as found in biofortified vitamin A maize, yellow cassava and orange-fleshed sweet potatoes. Millet was biofortified with iron.

The typical availability of biofortified crops in the market varied based on the crop and state (Table 1). In Enugu State, 89.47% of the biofortified maize was always available, and 100% of the biofortified millet was always available, while only 21.05% of the biofortified sweet potatoes were always available. The biofortified sweet potatoes in Enugu State were predominantly (63.16%) rarely available in the market. In Kaduna State, only 22.39% of the biofortified maize crops were reported as always available, and 70.15% were reported as rarely available.

On the other hand, the recorded non-biofortified crops were almost always available in the market (Table 1). In Enugu State, non-biofortified cassava and millet were always available in the market, 92.96% of the maize was always available and 75.25% of the sweet potatoes were always available. In Ogun State, non-biofortified cassava was always available, 63.63% of the maize was always available and 80% of the sweet potatoes were always available. In Kaduna State, none of these crops were recorded, suggesting that they were not available, at least at the time of data collection.

Insights from the key informant interviews explain some of the reasons for the limited availability of biofortified crops in the market. According to the key informant interviewees, biofortified crops have not been widely introduced in the open market in Nigeria yet. It was highlighted that the biofortified crop market is overseen by a set of biofortified seed providers, indicating that not everyone can participate and that biofortified seeds are not as readily available as other seeds. It was emphasized that biofortification to date has been a program-led intervention in Nigeria, working with a set of food producers and vendors. In addition, market dynamics play a role, as customers might not be aware of biofortified crops and therefore not demand or buy them, which in turn reduces their supply. It was noted that it is very challenging to increase the availability of biofortified crops unless there are specific government policies that, for example, could encourage farmers to produce more biofortified crops, as well as to invest in campaigns to increase customer awareness, which could subsequently improve the demand.

#### 3.1.2. Affordability

The market prices per crop were recorded in each state (Table 1). However, due to availability limitations, a price comparison between biofortified and non-biofortified crops was possible only in Enugu State. Namely, biofortified cassava, millet and sweet potato were more expensive than their non-biofortified equivalents: biofortified vitamin A cassava was 142% more expensive than non-biofortified cassava; biofortified orange-fleshed sweet potatoes were 98% more expensive than non-biofortified sweet potatoes; and iron pearl millet was 28% more expensive than normal millet. Notably, biofortified vitamin A maize was 29% cheaper than non-biofortified maize.

The key informant interviewees highlighted several factors contributing to the higher prices of biofortified crops. According to all interviewees, biofortified crops are considered premium products, and their higher prices might be due to the perceived nutritional benefits, limited market presence and middlemen who recognize the higher value of the crops and therefore increase the prices. It was noted that some of the biofortified crops, such as yellow cassava, which is visually different from white non-biofortified cassava, are associated in terms of visual similarities with local dishes that are a delicacy and are prepared only for special occasions. A key insight from the interviewees was that, in theory, the production prices of biofortified and non-biofortified crops are the same. In addition, it was highlighted that some biofortified crops, such as iron pearl millet, cost the same as their non-biofortified equivalents but yield larger amounts, which, could make the biofortified crop cheaper. Therefore, the higher prices are potentially driven by sellers’ prices and the perception of the crop as a premium product and limited demand and supply, rather than the costs associated with production.

### 3.2. Awareness, Perceptions, and Preferences

On average, 31.58% of the interviewed consumers were able to visually differentiate between biofortified and non-biofortified crops, and 14.30% of the consumers were aware of the crops being biofortified (Table 2). In Enugu State, 60% of the consumers were able to visually differentiate between the biofortified maize and orange-fleshed sweet potatoes and their non-biofortified equivalents, and less than half of them (20.83% for biofortified orange-fleshed sweet potato and 16.67% for biofortified maize) were aware that these crops were biofortified.

In Kaduna State, 17.16% of the consumers were aware of biofortified maize crops and 22.39% were able to visually differentiate between biofortified maize and non-biofortified maize. In Ogun State, the percentage of consumers who were aware of and able to differentiate biofortified cassava, maize and sweet potato from their non-biofortified ones was low, although, again, more consumers could visually differentiate the products without being aware of their biofortification and the potential benefits (11.53% and 21.15% for cassava, 2.08% and 22.92% for maize and 16.15% and 36.15% for sweet potatoes, respectively).

Insights from the key informant interviews indicate several reasons that consumers’ awareness is low. One is the fact that biofortification has been a program-led initiative, which limits the exposure of consumers who reside in areas where no such initiatives or educational campaigns have been conducted. An interviewee highlighted that just one education campaign is not worthwhile, and repetitive exposure to campaigns or messages about the benefits of biofortified crops is required. A key challenge is that conducting such educational campaigns can quickly become very expensive and resource management needs to be balanced.

With regard to preferences, 9.05% of the consumers indicated that they preferred biofortified crops to non-biofortified crops, 27.28% had no preference between the two groups and 64.64% preferred non-biofortified crops (Table 2). Between the states, there were some differences in preferences. In Enugu State, none of the interviewed consumers preferred biofortified cassava and millet, 21.11% preferred biofortified maize and 22.4% preferred biofortified sweet potato compared to their non-biofortified equivalents. In Ogun State, none of the consumers preferred biofortified cassava, less than 1% preferred biofortified maize and less than 2% preferred biofortified sweet potato compared to their non-biofortified equivalents. No customer preferences were recorded in Kaduna State.

The insights from the key informant interviews provide further details regarding why a larger percentage of consumers prefer non-biofortified crops. In contrast to large-scale fortification, where food vehicles are almost fully substituted for fortified ones (e.g., all salt to be iodized), in the case of biofortification, the consumer has to make an active choice between two products and thus might select the non-biofortified crop due to familiarity. Biofortified crops physically differ from their non-biofortified equivalents in terms of color, smell and taste; hence, behavioral change would be required to shift preferences. Furthermore, the interviewees suggested that the lack of uniform labeling, packaging or certification policy regarding biofortified crops and their benefits might also be associated with low consumer awareness and preferences.

### 3.3. Biofortified Crops and Nutrient Levels

**Figure 1:**
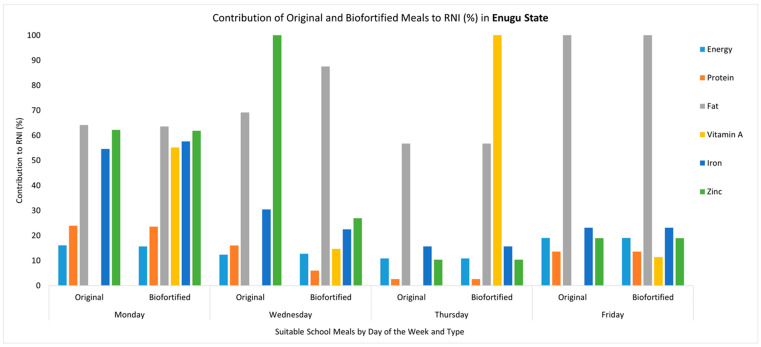
Comparing the micronutrient and macronutrient contributions of the original meals and modified meals that included a biofortified ingredient (biofortified) to the daily RNI (%) in Enugu State. Note: Values above 100% are capped; to see detailed estimates, refer to Table S1 in Supplementary Materials S2. The Tuesday meal was not suitable for modification with a biofortified ingredient. Energy is represented in g/kcal, protein in g/kcal, fat in g/kcal, vitamin A in mg/μg, iron in mg/μg and zinc in mg/μg. The recommended nutrient intake (RNI) is based on the following RNI values: energy = 1871 kcal, protein = 58 g, fat = 17 g, vitamin A = 475 µg, iron = 8 mg and zinc = 5 mg.

**Figure 2:**
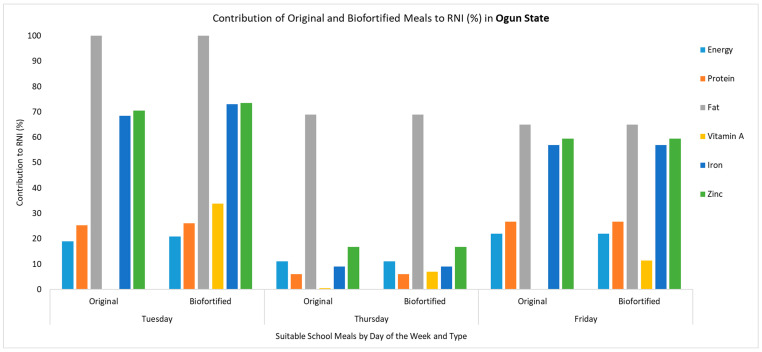
Comparing the micronutrient and macronutrient contributions of the original meals and the modified meals that included a biofortified ingredient (biofortified) to the daily RNI (%) in Ogun State. Note: Values above 100% are capped; to see detailed estimates, refer to Table S2 in Supplementary Materials S2. The Monday and Wednesday meals were not suitable for modification with a biofortified ingredient. Energy is represented in g/kcal, protein in g/kcal, fat in g/kcal, vitamin A in mg/μg, iron in mg/μg and zinc in mg/μg. The recommended nutrient intake (RNI) is based on the following RNI values: energy = 1871 kcal, protein = 58 g, fat = 17 g, vitamin A = 475 µg, iron = 8 mg and zinc = 5 mg.

**Figure 3:**
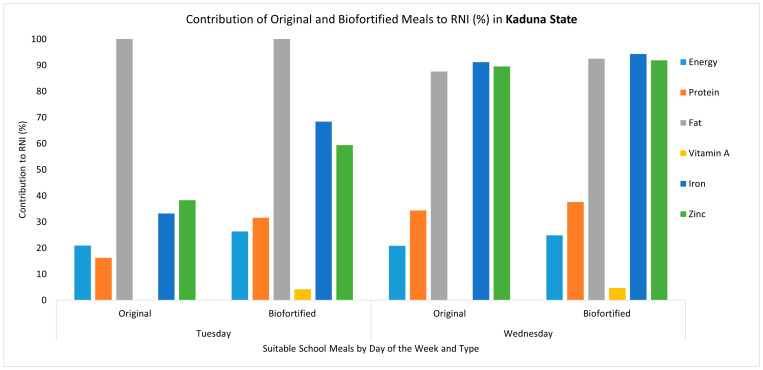
Comparing the micronutrient and macronutrient contributions of the original meals and the modified meals that included a biofortified ingredient (biofortified) to the daily RNI (%) in Kaduna State. Note: Values above 100% are capped; to see detailed estimates, refer to Table S3 in Supplementary Materials S2. The Monday, Thursday and Friday meals were not suitable for modification with a biofortified ingredient. Energy is represented in g/kcal, protein in g/kcal, fat in g/kcal, vitamin A in mg/μg, iron in mg/μg and zinc in mg/μg. The recommended nutrient intake (RNI) is based on the following RNI values: energy = 1871 kcal, protein = 58 g, fat = 17 g, vitamin A = 475 µg, iron = 8 mg and zinc = 5 mg.

The introduction of biofortified crops such as orange-fleshed sweet potatoes, vitamin A maize and vitamin A cassava in school meals led to increases in the content of vitamin A, zinc and iron. The original school meals did not contain vitamin A, contributing 0% to the daily RNI. When a biofortified ingredient was introduced, the content of vitamin A increased, contributing from 4% to 116% of the daily RNI. For example, the substitution of yam and sweet potato with biofortified orange-fleshed sweet potato in the Monday and Thursday meals in Enugu State increased the vitamin A content from 0 µg to 262.08 µg and from 0 to 548.80 µg, respectively, representing an increased contribution towards the daily RNI, which increased from 0% to 55.17% in the Monday meal and from 0% to 115.54% in the Thursday meal (Figure 1). Another example is the addition of orange-fleshed sweet potatoes in the Tuesday meal in Ogun State, where the vitamin A content increased from 0 µg to 160.16 µg (33.78% of the RNI) (Figure 2). However, other biofortified ingredients did not lead to large changes in the vitamin A content. For example, in Ogun State, the substitution of gari with vitamin A cassava gari in the Thursday meal led to a marginal increase in the vitamin A content from 2.55 µg to 33.15 µg (6.98% of the RNI) (Figure 2). Similarly, in Kaduna State, where a whole meal was replaced to include vitamin A maize and iron pearl millet, the vitamin A content only increased from 0 µg to 19.89 µg (4.19% of the RNI) (Figure 3). Nevertheless, due to the inclusion of iron pearl millet, there were significant increases in the iron and zinc content, each increasing from 2.66 mg to 5.47 mg (68.43% of RNI) and from 1.92 mg to 2.97 (59.39% of RNI), respectively (Figure 3). In addition, because this meal was fully replaced, the energy, protein and fat content also changed, with each being slightly higher than in the original meal. For the meals where the biofortified ingredient was substituted or added, there were no changes in the protein, fat and energy content of the meal. Detailed results regarding the estimated macro- and micronutrients for the original meals and the modified meals (where biofortified crops were introduced) for each state can be found in Supplementary Materials S2, Tables S4–S9.

#### Cost Implications

Due to the higher market prices of biofortified products, there will be cost implications for school meals. In Table 3, we provide the estimated prices of biofortified and non-biofortified crops as recorded in the field market survey. For example, biofortified orange-fleshed sweet potato is 98.13% more expensive than non-biofortified sweet potato.

The cost implications vary depending on how biofortified crops are integrated into the meals. Among the four different scenarios that we considered for the introduction of biofortified crops in school meals, the first option of substituting a non-biofortified crop with a biofortified equivalent is likely to be the cheapest, as no additional ingredients are required. However, due to the generally higher cost of biofortified crops, these meals are likely to be more expensive than the original meals. The second option of adding biofortified crops to the existing meal would likely be more expensive than the substitution option as the cost of the biofortified product would be added to the cost of the original meal and no product is removed. For meals that are entirely redesigned to include biofortified ingredients, estimating the cost implications becomes more complex, as all ingredients in the meal need to be changed; therefore, we cannot comment on whether they will be cheaper or more expensive in comparison to the original meal.

## 4. Discussion

Utilizing three different methodologies, we assessed the availability and affordability of biofortified crops, evaluated consumers’ and sellers’ awareness and preferences and simulated the potential impact on dietary quality if biofortified foods are introduced in predesigned and standardized school meals in three states in Nigeria.

Overall, we found that, out of all recorded crops, 39% of them were biofortified across the three states, with large inter-state variation. The most commonly available crop was biofortified vitamin A maize, and the least available was biofortified iron pearl millet. In Enugu State, all four biofortified crops released in Nigeria were observed, while, in Kaduna State, only biofortified vitamin A maize was recorded. The relatively low availability of the biofortified crops recorded in this study aligns with the consumption patterns documented in the National Food Consumption and Micronutrient Survey conducted in 2021, with 3%, 5% and 14% of the population consuming yellow cassava, orange-fleshed sweet potato and orange maize, respectively, in the last 30 days [6]. Insights from the key informant interviews indicate that the limited availability of biofortified crops could be due to limited uptake by farmers as biofortification is a program-led initiative. These differences are reflective of the regional variability overall and resources to conduct biofortification programs.

With regard to consumer awareness and preferences, we found that more than 30% of the consumers could visually differentiate biofortified from non-biofortified crops, 14% were aware that the crops were biofortified and less than 10% preferred biofortified options. The low consumer preference for biofortified crops found in this study is consistent with previous evidence from Nigeria, highlighting consumer acceptance as a key barrier due to the altered physical characteristics of biofortified crops [26,27,28,29]. Namely, taste acceptance studies indicate a lower preference for orange-fleshed sweet potato and yellow cassava due to color unsuitability in the preparation of local meals in Nigeria, and lighter-colored crop varieties were preferred [27,28,29]. Evidence from other countries shows that consumers’ acceptance of and preferences for biofortified crops were greatly improved through promotion and advertising strategies that emphasized the health and economic benefits of biofortified crops [26,29]. The low awareness of biofortified crops among the consumers found in this study suggests that there is a need for educational campaigns, which could improve both the awareness of and preferences for biofortified crops [26,29].

Nevertheless, this low acceptance could be due to challenges in incorporating biofortified foods into local meals. For example, in the simulation study, we were able to include biofortified foods only in 60% of the predesigned school meals. Moreover, for those in which inclusion was possible, their introduction was challenging. Namely, to align with local tastes and customs, crops in five meals (out of 15) were directly substituted with biofortified equivalents; the addition of biofortified crops was suitable for two meals, and, for two other meals, more than one ingredient had to be changed, thereby fully replacing the original school meal.

Even though biofortification as a process should not increase the prices of crops, we found that, in the market, biofortified crops were more expensive than their non-biofortified equivalent crops, which is in line with the previous literature [9,30]. Evidence from the key informant interviews highlights that the limited amount of biofortified crops makes them a premium product, and even though the cost of production might be the same as that of non-biofortified crops, the prices are higher due to the limited supply and demand. Therefore, even if the inclusion of biofortified foods in school meals leads to improvements in the vitamin A, iron and zinc content, this will be associated with cost implications, which need to be further investigated.

Notably, while biofortification could improve the nutrient content of school meals, in this study, we did not estimate the bioavailability of these nutrients. The bioavailability of micronutrients in biofortified foods can vary depending on the processing techniques. For example, vitamin A cassava retains moderate to high levels of provitamin A carotenoids when prepared using traditional African methods like boiling and frying [31,32]. Consumed daily in boiled form as a staple, it can meet 100% of young children’s average daily vitamin A requirements. However, when processed into fufu or chikwangue, as is commonly practiced in the Democratic Republic of Congo, or stored as gari (coarse flour) for extended periods, the retention significantly decreases [31,32]. This highlights the importance of local cooking practices and storage methods in determining the nutritional impact of biofortified crops. Future studies ought to investigate the bioavailability of the micronutrients in school meals using laboratory analyses [31,32].

A limitation of this study is that information regarding farmers’ acceptability and access to biofortified crops was not observed. Previous evidence indicates that the adoption of biofortified crops among farmers is very challenging and requires devoted extension services in order to educate farmers on new agricultural practices [33]. In addition, it has been found that farmers perceive biofortified seeds as more expensive to purchase and might require more effort to cultivate. There are critical issues around the availability of certified seeds, which are not unique to Nigeria. While more than 175 biofortified varieties of 13 staple crops have been released in 39 African countries, only a small fraction of these varieties are accessible to farmers [34]. Overall, institutional factors such as access to extension services, credit for purchasing seeds, cognitive factors such as farmers’ pre-existing perceptions and beliefs about biofortified crops and agronomic characteristics (yield, resistance to disease/pests) have been documented as key barriers to the adoption of biofortification by farmers [33]. While our study focused on the availability, affordability and consumer awareness of biofortified crops as observed in the market, we did not examine supply-side constraints such as agronomic challenges, seed accessibility or farmer incentives, which remain important factors influencing their adoption and warrant further investigation in future research. In addition, the generalizability of our findings is limited to the specific market locations included in our study. Future research incorporating household consumption studies or qualitative approaches across diverse settings could further complement and expand upon our findings.

## 5. Conclusions and Policy Implications

This study highlights that, in the presence of explicit national policy recognition of biofortification as a suitable intervention for MNDs and significant program interventions, the availability and consumer awareness of biofortified crops are low and the market prices of biofortified crops are high in the three studied states in Nigeria. While biofortified crops could offer nutritional benefits when integrated into standardized school meals, public health data suggest that, so far, this has not been effective in addressing diet quality at scale in Nigeria [6].

There are several key interrelated challenges around the availability of seeds, uptake by farmers, consumer awareness and high costs. While the development of new and better varieties is important, there needs to be a concerted policy, institutional response and resource allocation to integrate biofortified varieties into the food system. Our findings suggest that there is room for improvement among the different steps of the biofortification value chain [35]. Namely, to scale up biofortification, there is a need for targeted consumer awareness initiatives, which could improve the preferences and willingness to pay for biofortified crops, ultimately improving the demand and supply. Evidence suggests that initiatives targeted towards women could be especially impactful given their decision-making roles in food purchase and consumption [36]. In addition, the higher prices for biofortified crops found in this study suggest that it is crucial to implement regulatory oversight for the pricing and trading of biofortified crops to ensure that they can reach those who might need them most. Another area is related to improving the agricultural supply and farmers’ acceptance. For example, policy incentives, training and the sensitization of farmers through government extension support programs could encourage the uptake of certified biofortified varieties among farmers [37]. In addition, partnerships with private seed companies could improve the availability and accessibility of seeds [37]. Finally, introducing biofortified foods in school meals through local farmer groups could be an effective method to increase awareness among stakeholders and create demand, as demonstrated in the Home Grown School Feeding programs in Kenya, Tanzania and Malawi [38]. Future research ought to evaluate the specific programs and campaigns that could be suitable for the scaling up of biofortification in Nigeria.

## Figures and Tables

**Table 1:** Total number of recorded biofortified and non-biofortified crops per state, their typical availability in the market and their average market price in Nigerian Naira.

Crops per State	Biofortified Crops			Non-Biofortified Crops		
	Total Count	Typical Availability (Count)	Average Price	Total Count	Typical Availability (Count)	Average Price
Enugu						
Cassava	2	Rarely available (n = 2)	5500	8	Always available (n = 8)	2275
Maize	19	Always available (n = 17); only in season (n = 1); rarely available (n = 1)	2336.84	71	Always available (n = 66); rarely available (n = 5)	3289.86
Millet	3	Always available (n = 3)	3500	5	Always available (n = 5)	2740
Sweet Potato	19	Always available (n = 4); only in season (n = 3); rarely available (n = 12)	2889.47	101	Always available (n = 76); only in season (n = 18); rarely available (n = 7)	1457.91
Kaduna						
Cassava	0	na	na	0	na	na
Maize	134	Always available (n = 30); only in season (n = 9); rarely available (n = 94)	704.98	0	na	na
Millet	0	na	na	0	na	na
Sweet Potato	0	na	na	0	na	na
Ogun						
Cassava	0	na	na	52	Always available (n = 52)	5765.38
Maize	1	Only in season (n = 1)	400	143	Always available (n = 91); only in season (n = 49); rarely available (n = 3)	1493.71
Millet	0	na	na	0	na	na
Sweet Potato	0	na	na	130	Always available (n = 104); only in season (n = 22); rarely available (n = 4)	1158.46

Note: na stands for ‘not applicable’, as, for some fields, no information was available. The total count refers to the total number of biofortified and non-biofortified crops by type in each state, as recorded by the trained data collectors during the market visits. The typical availability refers to the seller’s answer to the question about the typical availability of the crops (sellers could select from the following 3 options: rarely available, always available and available only in season); refer to Supplementary Materials Section S1.1. The average price refers to the price of the crop per unit, as observed by the data collectors at the point of data collection.

**Table 2:** Percentage of consumers reporting being aware of biofortified crops, percentage of consumers able to visually differentiate between biofortified and non-biofortified crops and percentage of consumers preferring biofortified crops or non-biofortified crops and those without a preference.

Crops per State	Consumers					Sellers
	Awareness	Visual Difference	Consumer Preference			Visual Difference
	(% of consumers aware of biofortified crops)	(% of consumers able to differentiate between biofortified and non-biofortified crops)	(% of consumers preferring biofortified crops)	(% of consumers with no preference between biofortified crops and non-biofortified crops)	(% of consumers preferring non-biofortified crops)	(% of sellers able to differentiate between biofortified and non-biofortified products)
Enugu						
Cassava	30	30	30	0	70	30
Maize	16.67	60	21.11	21.11	57.78	65.55
Millet	0	0	0	37.5	62.5	0
Sweet Potato	20.83	60	10	22.4	67.5	67.5
Kaduna						
Maize	17.16	22.39	na	na	na	67.91
Ogun						
Cassava	11.54	21.15	0	23.08	76.92	42.31
Maize	2.08	22.92	0.69	43.05	63.19	36.11
Sweet Potato	16.15	36.15	1.53	43.85	54.61	45.38

Notes: na stands for ‘not applicable’, as, for some fields, no information was available. Values are presented as percentages (%) out of the total number of consumers and sellers that were interviewed. The values represent the answers to the questions asked by the trained data collectors (answers were recorded in binary yes/no); the field market questionnaire can be found in Supplementary Materials Section S1.1.

**Table 3:** The average prices of biofortified and non-biofortified crops per unit across the three states (represented in Nigerian Naira).

	Average Price	
Crop Type	Biofortified	Non-Biofortified
Cassava	5500	4020.19
Maize	1147.27	2391.78
Millet	3500	2740
Sweet Potato	2889.47	1308.18

Notes: The average price refers to the price of the crop per unit, as observed by the data collectors at the point of data collection, in Nigerian Naira. Prices were averaged across the states.

## Data Availability

The raw data from the field market study supporting the conclusions of this article will be made available by the authors on request. Data with regard to the school meals are provided in the Supplementary Materials.

## Supplementary Materials

The following supporting information can be downloaded at: https://www.mdpi.com/article/10.3390/nu17061036/s1, Section S1.1. Field Market Questionnaire. Section S1.2. Key Informant Interviews. Section S2. School Meals Simulation.

## Abbreviations

The following abbreviations are used in this manuscript:

MND	Macronutrient Deficiency
