# Supplementary material for: Availability, Affordability, Awareness, Preferences and Nutritional Impact of Biofortified Crops in Nigeria
Source: Nutrients. 2025 Mar 15;17(6):1036. doi: 10.3390/nu17061036 (PMC11946825; doi:10.3390/nu17061036)
Supplement: Supplementary file 1 [file nutrients-17-01036-s001.zip › nutrients-3517495-supplementary.pdf]

Supplementary Material for the following research article "*Availability, Affordability, Awareness, Preferences and Nutritional Impact of Biofortified Crops in Nigeria*"

Supplementary Materials S1

Supplementary S1.1

Field Market Questionnaire:

1. Crop type – write name.
2. Crop category – select from grains, tuber.
3. Biofortified nutrients – write down nutrients that crop is biofortified with.
4. Cost of crop per unit.
5. Is there a visual difference between biofortified and non-biofortified equivalent.
6. Can the consumer differentiate? – yes/no.
7. Is the consumer aware of biofortified crop? – yes/no.
8. Can the seller differentiate? – yes/no.
9. What does the consumer prefer – biofortified/non-biofortified/no preference.
10. When is the crop available in the market? – rarely available, always available, only in season.

Supplementary S1.2

Key Informant Interviews

Key Informant Interview 1 (TechnoServe) Questions Probed:

1. Could you provide some insights into the factors contributing to the higher prices of biofortified crops compared to their non-biofortified equivalents?
   - E.g. costs related to biofortification process or logistics?
2. Can you describe the supply chain logistics involved in obtaining biofortified seeds?
3. Are there challenges related to the storage of biofortified crops in Nigeria?
4. Could you elaborate on the government certification process for biofortified foods in Nigeria?

Key Informant Interview 2 (GAIN) Questions Probed:

1. Could you provide some insights into the factors contributing to the higher prices of biofortified foods compared to their non-biofortified equivalents?
   - Logistics, production.
2. What are the main challenges and barriers encountered in scaling up the production and distribution of biofortified crops across different states in Nigeria?
3. What are some of the main acceptability issues of biofortified crops in Nigeria? Would raising the awareness of the benefits associated with biofortified crops increase their demand?

4. Could you elaborate on the government certification process for biofortified foods in Nigeria?

Key Informant Interview 3 (HarvestPlus) Questions Probed:

1. What are the main reasons for the higher costs of biofortified foods compared to non-biofortified foods.
2. Challenges in biofortification.
3. Government policy around biofortification and certification process.
4. Questions about consumer acceptability- change in difference in taste, consistency that people might not like.

Supplementary Materials S2 – School Meals Simulation

Table S1. Predesign weekly school meals and their suitability for introducing biofortified foods in Enugu State. Handy measures and portion sizes are provided

Day	Type of Food	Food Item	Weight Per Food Item Per Child (G)	Bio-fortified food item for substitution
**Monday**	Yam and beans pottage with beef	Beans	38.3	
		Crayfish	1.0	
		Palm Oil	9.8	
		Yam	46.8	Orange fleshed sweet potato
		Beef	15	
**Tuesday**	Jollof rice with vegetable and chicken	Rice	97.2	Not suitable
		Chicken	18	
**Wednesday**	Okpa (Bambara nut) with dried fish	Okpa Flour	39	Vitamin A cassava
		Palm Oil	9.5	
		Crayfish	1.2	
		**Vitamin A cassava**	**98**	
**Thursday**	Fried sweet potato and stew	Sweet Potato	98	Orange fleshed sweet potato
		Palm oil	**9.5**	
**Friday**	Igba Oka (corn flour) mixed with black beans and vegetable	Oka flour	46.2	Vitamin A cassava flour
		Crayfish	1.0	
		Palm Oil	15	
		Black beans	11.2	

Table S2. Predesign weekly school meals and their suitability for introducing biofortified foods in Ogun State. Handy measures and portion sizes are provided.

**Day**	**Type of Food**	**Food Item**	**Weight Per Food Item Per Child**	**Bio-fortified food item for substitution**
**Monday**	Rice and Beans with vegetable and fish	Rice	45	Not suitable
		Beans	32	
		Palm oil	9.0	
		Fish	7.5	
**Tuesday**	Beans porridge and fish	Beans	57.2	Orange flesh sweet Potato
		Palm Oil	17.5	
		Fish	4.1	
**Wednesday**	Rice, Vegetable soup and Meat	Rice	55	Not suitable
		Palm Oil	9.0	
		Meat	15	
**Thursday**	Eba, Vegetable and Meat	Gari	25.5	Vitamin A cassava
		Meat	15	
		Palm Oil	10.7	
**Friday**	Eko (Corn meal) and Moimoi (Beans puddy)	Cornmeal	Aggregated	Vitamin A maize
		Beans	45.8	
		Palm Oil	8.4	

Table S3. Predesign weekly school meals and their suitability for introducing biofortified foods in Kaduna State. Handy measures and portion sizes are provided.

Day	Type of Food	Food Item	Weight Per Food Item Per Child	Bio-fortified food item for inclusion
**Monday**	Moi Moi with vegetable	Beans	45.8	Not suitable
		Crayfish	0.75	
		Palm Oil	8.4	
**Tuesday**	Jollof Rice & Beans	Rice	45	Iron Pearl Millet, Vitamin A maize, Soya, maize, millet, groundnuts
		Beans	22	
		Palm Oil	17	
		Crayfish	0.75	
**Wednesday**	Beans Porridge	Beans	76.4	Vitamin A maize
		Crayfish	2.5	
		Palm Oil	13.7	
**Thursday**	Jollof Rice&Egg	Rice	97.2	Not suitable
		Crayfish	0.65	
		Palm Oil	14	
		Egg	Table sized egg	
**Friday**	Rice&G/Nut Soup	Rice	120	Not Suitable
		Palm Oil	4	
		Crayfish	2	
		Groundnut flour	18	

Table S4. Nutritional Content of the Original School Meals Menu in Enugu State. Highlighted products are the ones that were substituted.

Day	Type of Food	Food Item	Weight Per Food Item Per Child (G)	Energy (g/kcal)	Protein (g/kcal)	Fat (g/kcal)	Vitamin A (mg/ug)	Iron (mg/ug)	Zinc (mg/ug)
**Monday**	Yam and beans pottage with beef	Beans	38.3	130.24	9.17	0.56	0.00	3.30	2.24
		Crayfish	1	3.33	0.67	0.05	0.00	0.29	0.00
		Palm Oil	9.8	87.71	0.00	9.75	0.00	0.00	0.00
		Yam	46.8	65.50	0.94	0.19	0.00	0.36	0.27
		Beef	15	15.48	3.09	0.35	0.00	0.42	0.60
Total Macro and Micro Nutrients				302.26	13.87	10.90	0.00	4.37	3.11
RNI				1871.00	58.00	17.00	475.00	8.00	5.00
RNI(%)				16.16	23.92	64.11	0.00	54.59	62.19
**Wednesday**	Okpa (Bambara nut) with dried fish	Okpa Flour	39	143.70	8.62	2.26	0.00	2.15	5.00
		Palm Oil	9.5	85.03	0.00	9.45	0.00	0.00	0.00
		Crayfish	1.2	3.33	0.67	0.05	0.00	0.29	0.00

 https://doi.org/10.3390/nu17061036

Total Macro and Micro Nutrients				232.06	9.29	11.76	0.00	2.44	5.00
RNI				1871.00	58.00	17.00	475.00	8.00	5.00
RNI(%)				12.40	16.02	69.18	0.00	30.45	100.03

**Thursday**	Fried sweet potato and stew	Sweet Potato	98	118.58	1.52	0.20	0.00	1.25	0.52
		Palm oil	9.5	85.03	0.00	9.45	0.00	0.00	0.00
Total Macro and Micro Nutrients				203.61	1.52	9.64	0.00	1.26	0.52
RNI				1871.00	58.00	17.00	475.00	8.00	5.00
RNI(%)				10.88	2.62	56.73	0.00	15.69	10.39
**Friday**	Igba Oka (corn flour) mixed with black beans and vegetable	Oka flour	46.2	180.5311	4.53068	2.02356	0	0.6006	0.29106
		Crayfish	1	3.33	0.67	0.05	0.00	0.29	0.00
		Palm Oil	15	134.26	0.00	14.92	0.00	0.00	0.00
		Black beans	11.2	38.09	2.68	0.17	0.00	0.96	0.66
Total Macro and Micro Nutrients				356.20	7.89	17.16	0.00	1.85	0.95
RNI				1871.00	58.00	17.00	475.00	8.00	5.00
RNI(%)				19.04	13.60	100.95	0.00	23.16	18.96

Table S5. Nutritional Content of the Amended School Meals to Include Biofortification in Enugu State. Highlighted products are the ones that were included.

Day	Type of Food	Food Item	Weight Per Food Item Per Child (G)	Energy (g/kcal)	Protein (g/kcal)	Fat (g/kcal)	Vitamin A (mg/ug)	Iron (mg/ug)	Zinc (mg/ug)
**Monday**	Yam and beans pottage with beef	Beans	38.3	130.24	9.17	0.56	0.00	3.30	2.24
		Crayfish	1	3.33	0.67	0.05	0.00	0.29	0.00
		Palm Oil	9.8	87.71	0.00	9.75	0.00	0.00	0.00
		Orange Fleshed Sweet Potato	46.8	56.63	0.73	0.09	262.08	0.60	0.25
		Beef	15	15.48	3.09	0.35	0.00	0.42	0.60
Total Macro and Micro Nutrients				293.38	13.66	10.81	262.08	4.61	3.09
RNI				1871.00	58.00	17.00	475.00	8.00	5.00
RNI(%)				15.68	23.56	63.56	55.17	57.62	61.83
**Wednesday**	Abacha	Palm Oil	9.5	85.03	0.00	9.45	0.00	0.00	0.00
		Crayfish	1.2	3.33	0.67	0.05	0.00	0.29	0.00
		Garden eggs	10	3.50	0.16	0.02	0.00	0.21	0.35
		Vitamin A Cassava	58	84.88	1.10	0.46	69.60	0.72	0.88
		Garden egg leaves	1	0.32	0.04	0.01	0.00	0.02	0.02

		African oil bean seed	10	61.74	1.50	4.89	0.00	0.56	0.10
Total Macro and Micro Nutrients				238.80	3.47	14.88	69.60	1.80	1.35
RNI				1871.00	58.00	17.00	475.00	8.00	5.00
RNI(%)				12.76	5.99	87.52	14.65	22.51	26.98
**Thursday**	Fried sweet potato and stew	Orange Fleshed Sweet Potato	98	118.58	1.52	0.20	548.80	1.25	0.52
		Palm oil	9.5	85.03	0.00	9.45	0.00	0.00	0.00
Total Macro and Micro Nutrients				203.61	1.52	9.64	548.80	1.26	0.52
RNI				1871.00	58.00	17.00	475.00	8.00	5.00
RNI(%)				10.88	2.62	56.73	115.54	15.69	10.39
**Friday**	Igba Oka (corn flour) mixed with black beans and vegetable	Vitamin A Maize	46.2	180.5311	4.53068	2.02356	54.054	0.6006	0.29106
		Crayfish	1	3.33	0.67	0.05	0.00	0.29	0.00
		Palm Oil	15	134.26	0.00	14.92	0.00	0.00	0.00
		Black beans	11.2	38.09	2.68	0.17	0.00	0.96	0.66
Total Macro and Micro Nutrients				356.20	7.89	17.16	54.05	1.85	0.95
RNI				1871.00	58.00	17.00	475.00	8.00	5.00
RNI(%)				19.04	13.60	100.95	11.38	23.16	18.96

Table S6. Nutritional Content of the Original School Meals Menu in Ogun State. Highlighted products are the ones that were substituted.

Day	Type of Food	Food Item	Weight Per Food Item Per Child (G)	Energy (g/kcal)	Protein (g/kcal)	Fat (g/kcal)	Vitamin A (mg/ug)	Iron (mg/ug)	Zinc (mg/ug)
**Tuesday**	Beans porridge and fish	Beans	57.2	194.51	13.70	0.84	0.00	4.93	3.35
		Palm Oil	17.5	156.63	0.00	17.40	0.00	0.00	0.00
		Fish	4.1	5.1168	1.02705	0.1066	0.3075	0.54612	0.17794
Total Macro and Micro Nutrients				356.26	14.73	18.35	0.31	5.47	3.53
RNI				1871.00	58.00	17.00	475.00	8.00	5.00
RNI(%)				19.04	25.39	107.96	0.06	68.43	70.52
**Thursday**	Eba, Vegetable and Meat	Gari	25.5	92.61313	0.407044	0.184556	0	0.5525	0.6375
		Meat	15	20.205	3.06	0.885	2.55	0.165	0.204
		Palm Oil	10.7	95.77	0.00	10.64	0.00	0.00	0.00
Total Macro and Micro Nutrients				208.59	3.47	11.71	2.55	0.72	0.84
RNI				1871.00	58.00	17.00	475.00	8.00	5.00

RNI(%)				11.15	5.98	68.89	0.54	8.98	16.83
**Friday**	Eko (Corn meal) and Moimoi (Beans puddy)	Cornmeal	46.2	180.5311	4.53068	2.02356	0	0.6006	0.29106
		Beans	45.8	155.74	10.97	0.68	0.00	3.94	2.68
		Palm Oil	8.4	75.18	0.00	8.35	0.00	0.00	0.00
Total Macro and Micro Nutrients				411.46	15.50	11.05	0.00	4.55	2.97
RNI				1871.00	58.00	17.00	475.00	8.00	5.00
RNI(%)				21.99	26.73	65.01	0.00	56.83	59.44

Table S7. Nutritional Content of the Original School Meals Menu in Ogun State. Highlighted products are the ones that were substituted.

Day	Type of Food	Food Item	Weight Per Food Item Per Child (G)	Energy (g/kcal)	Protein (g/kcal)	Fat (g/kcal)	Vitamin A (mg/ug)	Iron (mg/ug)	Zinc (mg/ug)
**Tuesday**	Beans porridge and fish	Beans	57.2	194.51	13.70	0.84	0.00	4.93	3.35
		Orange Fleshed Sweet Potato	28.6	34.61	0.44	0.06	160.16	0.37	0.15
		Palm Oil	17.5	156.63	0.00	17.40	0.00	0.00	0.00
		Fish	4.1	5.1168	1.02705	0.1066	0.3075	0.54612	0.17794
Total Macro and Micro Nutrients				390.86	15.17	18.41	160.47	5.84	3.68
RNI				1871.00	58.00	17.00	475.00	8.00	5.00
RNI(%)				20.89	26.16	108.30	33.78	73.01	73.55
**Thursday**	Eba, Vegetable and Meat	Gari - Vitamin A cassava	25.5	92.61313	0.407044	0.184556	30.6	0.5525	0.6375
		Meat	15	20.205	3.06	0.885	2.55	0.165	0.204
		Palm Oil	10.7	95.77	0.00	10.64	0.00	0.00	0.00
Total Macro and Micro Nutrients				208.59	3.47	11.71	33.15	0.72	0.84
RNI				1871.00	58.00	17.00	475.00	8.00	5.00

RNI(%)				11.15	5.98	68.89	6.98	8.98	16.83

**Friday**	Eko (Corn meal) and Moimoi (Beans puddy)	Vitamin A Maize	46.2	180.5311	4.53068	2.02356	54.054	0.6006	0.29106
		Beans	45.8	155.74	10.97	0.68	0.00	3.94	2.68
		Palm Oil	8.4	75.18	0.00	8.35	0.00	0.00	0.00
Total Macro and Micro Nutrients				411.46	15.50	11.05	54.05	4.55	2.97
RNI				1871.00	58.00	17.00	475.00	8.00	5.00
RNI(%)				21.99	26.73	65.01	11.38	56.83	59.44

Table S8. Nutritional Content of the Amended School Meals to Include Biofortification in Kaduna State. Highlighted products are the ones that were included.

**Day**	**Type of Food**	**Food Item**	**Weight Per Food Item Per Child (G)**	**Energy (g/kcal)**	**Protein (g/kcal)**	**Fat (g/kcal)**	**Vitamin A (mg/ug)**	**Iron (mg/ug)**	**Zinc (mg/ug)**
**Tuesday**	Jollof Rice & Beans	Rice	45	162.988714	3.67875	0.71485714	0	0.55005	0.62775
		Beans	22	74.81	5.27	0.32	0.00	1.89	1.29
		Palm Oil	17	152.16	0.00	16.91	0.00	0.00	0.00
		Crayfish	0.75	2.49	0.50	0.04	0.00	0.21	0.00
Total Macro and Micro Nutrients				392.45	9.45	17.99	0.00	2.66	1.92
RNI				1871.00	58.00	17.00	475.00	8.00	5.00
RNI(%)				20.98	16.30	105.80	0.00	33.26	38.33
**Wednesday**	Beans Porridge	Beans	76.4	259.79	18.30	1.13	0.00	6.58	4.47
		Crayfish	2.5	8.31	1.68	0.14	0.00	0.72	0.00
		Palm Oil	13.7	122.62	0.00	13.62	0.00	0.00	0.00

Total Macro and Micro Nutrients				390.73	19.98	14.89	0.00	7.30	4.48
RNI				1871.00	58.00	17.00	475.00	8.00	5.00
RNI(%)				20.88	34.45	87.57	0.00	91.21	89.52

Table S9. Nutritional Content of the Original School Meals Menu in Kaduna State. Highlighted products are the ones that were substituted.

Day	Type of Food	Food Item	Weight Per Food Item Per Child (G)	Energy (g/kcal)	Protein (g/kcal)	Fat (g/kcal)	Vitamin A (mg/ug)	Iron (mg/ug)	Zinc (mg/ug)
**Tuesday**	Maize & Beans	Vitamin A Maize	17	66.4292	1.667133	0.7446	19.89	0.221	0.1071
		Beans	22	74.81	5.27	0.32	0.00	1.89	1.29
		Palm Oil	17	152.16	0.00	16.91	0.00	0.00	0.00
		Crayfish	0.75	2.49	0.50	0.04	0.00	0.21	0.00
		Iron Pearl Millet	17	60.01765	1.56655	0.75905	0	1.309	0.4811
		Soya	17	72.8161	5.9279	2.9495	0	1.27262	0.68595
		Groundnuts	17	64.787	3.4	1.105	0	0.561	0.4063
Total Macro and Micro Nutrients				493.51	18.34	22.83	19.89	5.47	2.97
RNI				1871.00	58.00	17.00	475.00	8.00	5.00
RNI(%)				26.38	31.61	134.29	4.19	68.43	59.39
**Wednesday**	Beans Porridge	Beans	76.4	259.79	18.30	1.13	0.00	6.58	4.47
		Crayfish	2.5	8.31	1.68	0.14	0.00	0.72	0.00
		Palm Oil	13.7	122.62	0.00	13.62	0.00	0.00	0.00
		Vitamin A Maize	19.1	74.63516	1.873073	0.83658	22.347	0.2483	0.12033

Total Macro and Micro Nutrients				465.36	21.86	15.72	22.35	7.55	4.60
RNI				1871.00	58.00	17.00	475.00	8.00	5.00
RNI(%)				24.87	37.68	92.49	4.70	94.32	91.93
